# Dimensional Changes of the Soft Tissue after Alveolar Ridge Preservation with a Collagen Material. A Clinical Randomized Trial

**DOI:** 10.2174/1874210601812010389

**Published:** 2018-05-30

**Authors:** Sigmar Schnutenhaus, Thomas Martin, Jens Dreyhaupt, Heike Rudolph, Ralph G. Luthardt

**Affiliations:** 1 Private practice, Breiter Wasmen 10, D-78247, Hilzingen, Germany; 2 Department of Prosthetic Dentistry, Center of Dentistry, University of Ulm, Ulm, Germany; 3 Private practice, Füssen, Germany; 4 Institute of Epidemiology and Medical Biometry, University of Ulm, Ulm, Germany

**Keywords:** Alveolar bone loss, Alveolar Process, Bone Regeneration, Bone substitute, Collagen, Tooth extraction, Soft Tissue

## Abstract

**Background:**

Reduction of the soft tissue is an unavoidable consequence of tooth extraction without appropriate measures of Alveolar Ridge Preservation (ARP).

**Objectives:**

The objective of this study is the volumetric investigation of the dimensional change of the soft tissue post tooth extraction to compare an Alveolar Ridge Preservation (ARP) measure with the insertion of a combination material with a collagen cone to fill the alveolus, combined with a collagen membrane, with untreated extraction alveoli.

**Methods:**

In the context of a randomized clinical trial, 31 patients were treated with the combination material directly post tooth extraction in the maxilla (ARP). In 29 further patients, the extraction alveoli were left without further measures (control group).

The changes of the soft tissue contour were measured 6 (+/- 1) weeks post extraction. The measurements were performed by superimposing digital models. The groups were compared using the Wilcoxon rank-sum-test.

**Results:**

The premolar subgroup revealed a significant difference of the soft tissue dimension post insertion of a collagen material into the alveolus in comparison to untreated alveoli. In these cases, the mean loss of soft tissue volume after use of the collagen material was significantly lower.

**Conclusion:**

The proposed hypothesis that there is a difference of the soft tissue preservation between alveoli with and without the use of a collagen material can be accepted with restrictions to the premolar region. A statistically significant lower volume reduction of the soft tissue by implantation of the collagen material could be detected with premolars.

## 1 INTRODUCTION

There are changes in the resorption characteristics of the alveolar process post tooth extractions [1]. Specifically, there is even initially a significant resorption in the buccal portion of the cavity, which eventually results in a loss of volume of the alveolar process [2]. The position, angulation and, consequently, the prognosis of the implant are significantly affected by the available volume of hard and soft tissue [3]. The natural regeneration post tooth loss starts with a blood filling defect; this forms a stable blood clot, which is then overgrown by the epithelium and facilitates the internal bone regeneration [4]. Within the blood clot, fibrin forms a natural support structure and scaffold, which facilitates the formation of osteoid and its subsequent calcification [4]. This bone regeneration is completed after approximately 120 days; the periosteum has fully stabilized after approximately 180 days [5, 6]. However, the remodeling processes during the bone regeneration from connective tissue to mineralize new bone occur over periods that vary widely between individuals and are not predictable [7]. Thus, there is a mean horizontal degeneration of the alveolar process of 3.8 mm and a mean vertical degeneration of 1.2 mm over the first six months post extraction [8, 9]. In this process, the vestibular degeneration is significantly more pronounced, which could be due to a reduced blood supply to the thin vestibular bone [10]. Approaches to stabilize the bone and thus reduce the resorption processes include the insertion of materials into the alveolar void as a measure for alveolar ridge preservation (ARP) [11, 12]. Apart from autologous bone, allogenic, xenogeneic or synthetic bone replacement materials are available for ARP. The different ARP measures bring about a reduction of the dimensional change of the hard and soft tissue, but cannot prevent resorption entirely [13]. Apart from bony regeneration, the preservation of adequate soft tissue around the implant has functional and aesthetic significance [14]. A study with a bovine bone replacement material revealed no significant differences to the soft tissue progression after ten years after filling an alveolus with this material and covering with a membrane in terms of a guided bone regeneration [GBR] with delayed implantation [15]. The filling of the alveolar void with a bone replacement material could play a supporting role for the soft tissue. However, there is no evidence available for this to date [16].

A further, intra-individual comparator study by Barone *et al*. determined better conditions in terms of bone and soft tissue preservation post-ARP with the porcine material [17]. Measures of ARP may improve any gain of keratinized gingiva and thus improve the aesthetic and functional outcome [17]. The long-term success of preservation with implanted prosthetics is influenced by a wide range of extraneous factors [18]. The preservation of hard and soft tissue and the correct implant position play an important role in this [19, 20]. Scientific data appear to indicate that an adequate width of attached mucosa may facilitate oral hygiene procedures thus preventing peri-implant inflammation and tissue breakdown [21]. At the moment, there is insufficient reliable evidence to provide recommendations for the best soft tissue augmentation technique that whether techniques to increase the width of keratinised/attached mucosa are beneficial to patients or not, and which are the best incision/suture techniques/materials [22]. Clinical trials on the efficacy of soft tissue augmentation procedures around dental implants show an increase in soft tissue thickness by using autogenous subepithelial connective tissue grafts [23].

A reliable and promising method for reducing soft tissue contour loss by applying a fully resorbable material may thus be of significance for the clinical outcome. A fully resorbable material is available in the form of a combination material consisting of a collagen cone with a proportion of equine collagen fibrils of 32.2 mg and an equine collagen membrane without chemical cross-linking (PARASORB Sombrero^®^ (Resorba, Nuremberg, Germany)). Both materials are combined in one product to facilitate a simple and fast application. So far, there are no adequate clinical human studies available for this material [24]. A systematic literature search about the use of pure collagen materials for ARP with the search term: (Clinical and Trial Study or Systematic Review) AND (ARP or “Alveolar Ridge Preservation” or “Socket Preservation”) OR (Tooth or Teeth and Ridge Preservation or Socket Preservation) AND collagen*) revealed no indications for clinical efficacy of ARP with a collagen cone for the filling of the alveolus in combination with a collagen membrane to cover the extraction wound.

The objective of this study is the volumetric investigation of the clinical application of the combination material for the preservation of soft tissue in comparison to untreated extraction alveoli. The proposed hypothesis is that there is a difference in the preserved soft tissue between the alveoli with and without application of a combination material consisting of a collagen cone and a collagen membrane.

## 2 MATERIALS AND METHODS

The study was performed as a monocenter, prospective and randomized clinical study in accordance with the Declaration of Helsinki. The procedure and all materials used were submitted to the Central Ethics Committee and approved by the committee (Ethics Committee of Ulm University, application no. 337/12, approved on 02/13/2013). The study was registered in the German Clinical Trial Register and the International Clinical Trials Registry Platform of the WHO as DRKS 00004769, date of registration: Feb. 28, 2013; and DRKS00005978, date of registration: Nov. 09, 2015.

The study subjects were educated both verbally and in writing and have provided their written consent before participation in the study.

### 2.1 Study Patients

Sixty patients participated in the study, who required removal of a tooth from the maxilla due to periodontal disease or due to the destruction of a tooth by caries or trauma. It was a requirement that the subsequent closure of the gap was to be performed with a fixed implant-supported crown. The patient's consent was needed for the replacement of missing tooth by an implant available prior to enrollment in the study.

It was required for enrollment in the patient group that a tooth or existing implant remained in place directly adjacent to the tooth to be extracted. A further requirement was compliance with all of the following criteria:

- Age above 18 years. The participants had to be legally competent. No detectable primary additional augmentation required, due to advanced vertical bone defects.
- Non-smoker and/or no more than 10 cigarettes/day.
- No administration of bisphosphonates.
- No pregnancy.
- No alcohol or drug abuse.
- No infectious disease, such as hepatitis or HIV and/or AIDS.
- No uncontrolled severe diabetes mellitus. The long-term blood glucose HbA1c level must be below 6.7%.

### 2.2 Treatment Protocol

All measures (interventions) and the follow-up assessments were performed in the practice of the primary author. All patients were treated by SiS only.

The local anesthesia was performed with Ultracain DS 1:200,000 (sanofi aventis, Frankfurt, Germany). For molars, the crown was decapitated and the roots separated using a diamond burs drill with the dental turbine. The atraumatic extraction was performed using periotomes and removal of the tooth with dental forceps after complete mobilization. The extraction alveolus was then subjected to careful curettage.

No further measures were then performed on the alveolus in the control group.

In der ARP group, the collagen material was inserted in accordance with the manufacturer's information. A circular supraperiosteal pocket of the coronal soft tissue was prepared. The soft tissue was not mobilized during this process and therefore, the alveolus was not primarily closed by mucosa. The collagen cone that was trimmed to the size of the alveolus and the trimmed membrane was inserted into the alveolus without pressure. Where teeth had multiple roots, the collagen cone was divided into shapes that matched the root, in accordance with the anatomy of the root. A cross mattress suture was applied using monofilament Resolon 4/0 polyamide-6 suture material (Resorba, Nuremberg, Germany) to protect the cone from exposure beyond the alveolus. The wound was visually inspected in all patients after one week. At that time, the suture was removed in the patients in the ARP group.

After the extraction, all patients received instructions on how to behave in the next 24 hours. These included:

Instruction not to eat while the anesthetic effect persisted

Complete abstinence from alcohol, coffee or caffeinated drinks and cigarettes or other smoking products

Instruction not to rinse the extraction wound to maintain the blood clot

**Fig. (1):**
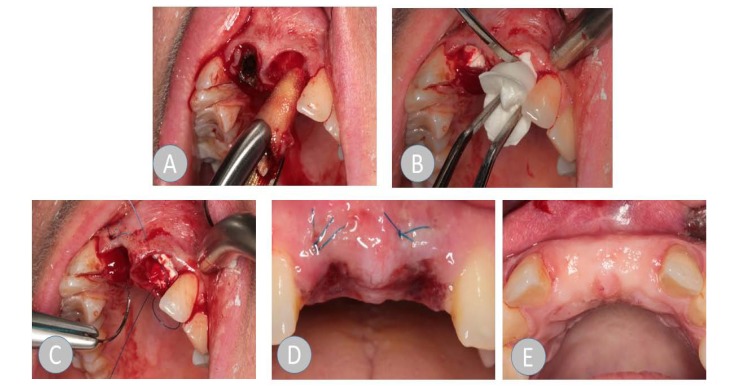
Surgical procedures of the insertion of the collagen combination material. (A) Atraumatic tooth extraction with periotomes and forceps. (B) Introduction of the collagen cone with membrane (C) Temporary sutures to stabilize the membrane. (D) Status after seven days at the time of suture removal (E) Status after three months at the time of the implantation.

No manual manipulation of the wound (pulling the lip, massive cleaning of the wound, *etc*.) (Fig. **1**).

The patients had 600 mg Ibuprofen prescribed for pain reduction. This was administered as required by each patient. A prophylactic antibiotic was not prescribed.

A provisional interim prosthetic was applied in exceptional cases only (aesthetics of the front teeth or function where multiple teeth were lost) and only at the patient's request.

The implant was fitted after 11 (+/- 1) weeks.

### 2.3 Objectives and Data Acquisition

The objective was to determine the reduction of the volume in the area of the extraction alveolus. The soft tissue was inspected at the time of the extraction (T0) and after a healing time of 6 (+/- 1) weeks (T1). In the data presented here, only the outer contour of the soft tissue was examined, without considering the situation of the bone in the area of the former alveolus.

An impression was taken with alginate immediately post extraction (Blueprint cremix, Dentsply DeTrey, Constance, Germany). The alginate was mixed in line with manufacturer's instructions and filled in prefabricated closed metal impression trays.

At time T1, a precision impression was taken with a polyether impression material (Permadyne Garant 3M Espe, Seefeld, Germany). This impression was used to produce an X-ray template. At the same time, the model thus obtained provided the basis for this data analysis.

### 2.4 Digitalization of the Plaster Models

Both impressions were used to produce models from a special plaster suitable for digitalization HS-CAD/CAM (Henry Schein Inc., Melville, NY, USA). This model was digitalized using a model scanner (3Shape Scanner D 700, 3Shape A/S, Copenhagen, Denmark) with a measurement uncertainty of ±16 µm according to a standardized measurement plan and represented as Stereolithography (STL) surfaces.

### 2.5 Creation of Surface Models, Reprocessing and Matching of the Data

These surface models were superimposed on the computer to analyze the change of the soft tissue surrounding the extraction alveoli and thus the differences before and after extraction were measured.

**Fig. (2):**
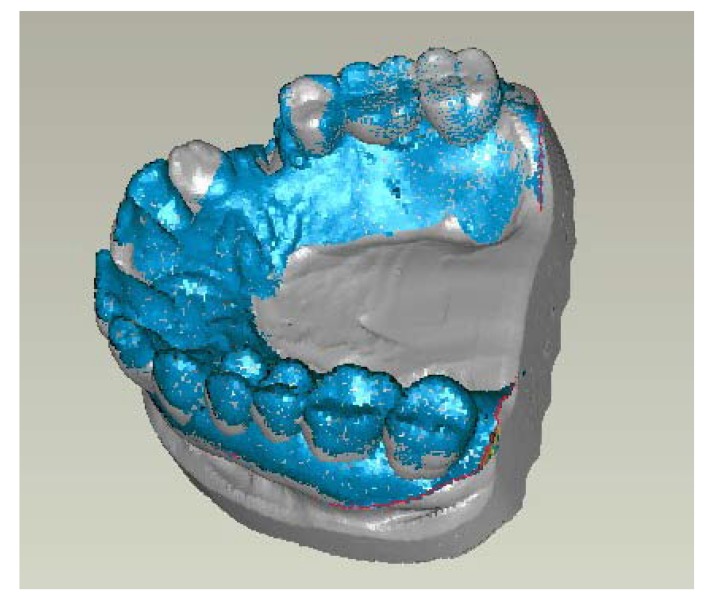
Superimposition of the models at time T0 (immediately after extraction) and T1 (6 (+/-1) weeks) *via* the hard tooth tissue. The generated model datasets were superimposed using Geomagic Studio software (geomagic studio 9 and qualify 9, geomagic, Research Triangle Park, NC, USA).

Reference structures were defined for the exact superimposition of the model. Then matching took place *via* the hard tooth tissue (Fig. **2**). The generated model datasets were superimposed using Geomagic Studio software (geomagic studio 9 and qualify 9, geomagic, Research Triangle Park, NC, USA). It can be assumed that the matching *via* the hard tooth tissue is confirmed in view of the short observation window of 6 (+/- 1) weeks, because almost no changes were expected by abrasion, for example, in that area.

In the subsequent step, the Region of Interest (ROI), *i.e*. the soft tissue surrounding the extraction alveoli, was defined. The digitalized models (T0 and T1) were reduced accordingly. The ROI was defined as follows:

- In the mesiodistal orientation up to the adjacent teeth.
- Vestibular to a maximum height of the top of the vestibule (depending on the recorded data and/or the information captured in the impression).
- Palatinal in the same vertical height as vestibular, as a maximum up to the lowest point of the captured palate.
- At the extraction alveoli themselves, starting at the maximum point of the gingival margin surrounding the alveoli.

### 2.6 3D Measurement

**Fig. (3):**
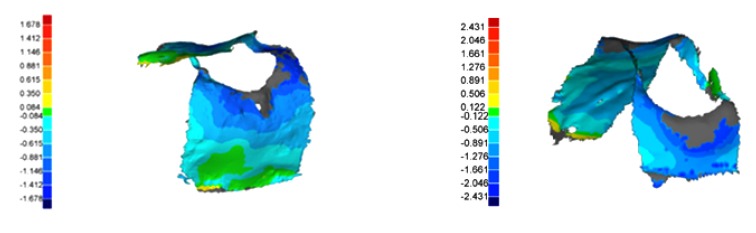
Analysis of the soft tissue changes in the region of the extraction alveolus. The matched model datasets were measured in three dimensions. The three-dimensional analysis method requires a separation into positive and negative measurement points.

The matched model datasets were measured in three dimensions using Geomagic Studio software (geomagic qualify 9, geomagic, Research Triangle Parc, NC, USA) (Fig. **3**). The three-dimensional analysis method requires a separation into positive and negative measurement points. Only the negative values were included in the analysis because the positive values must be treated as artifacts.

### 2.7 Case Number Count

Due to a lack of clinical data, case numbers could not be estimated in advance. Therefore, this study was performed as an explorative study.

### 2.8 Randomization

A randomization list was produced for the entire study with 60 patients (Institute of Epidemiology and Medical Biometry, Ulm University, Germany), where the patients were assigned to the respective group in six strata. The data were stratified by

- Gender (two groups: male/female), and
- Region of the studied tooth (three groups: anterior teeth/premolar/molar).

The principal investigator or an individual authorized by him/her assigned the therapy form to the treatment center by fax according to the randomization list.

### 2.9 Blinding

The STL datasets of the situations at times T0 and T1 were forwarded to the analyst (TM) in blinded and anonymized form. Blinding was performed only after completion of the analysis, documentation and statistical analysis. The deblinding was performed locally and by different individuals than those involved in the analysis.

### 2.10 Statistical Analysis

Minimum, median and maximum were reported for the metric target parameters. Differences between the test and control group were reviewed using the Wilcoxon rank-sum test. Due to the explorative character of the study, all outcomes from the statistical test must be interpreted as generating hypotheses and not as proof. All statistical tests were performed at an alpha = 0.05 significance level (two-tailed). There was no adjustment for multiple testing.

## 3 RESULTS

All patients were treated according to the clinical protocol. There were no postoperative complications. All enrolled patients completed the study.

Thirty-one patients were treated with the collagen by stratified randomization by gender and tooth region. Twenty-nine patients formed the control group with extraction without further concomitant measures. Thirty-one female and 29 male patients participated in the study. The ARP group included 15 male and 16 female patients; the control group comprised 14 male and 15 female subjects. The mean patient age was 52.3 years (24 - 78 years). The randomized distribution of the teeth is illustrated in Table **1**.

The explorative data analysis reflects the negative maxima, the negative medians and the standard deviations. Since there was no normal distribution, the *p*-values were determined using the Wilcoxon rank-sum test (Table **2**). There were no significant differences detected in any of the analyses.

This was followed by an analysis of the individual tooth groups. Due to the small sample size, the molars were not considered. The review of the individual tooth regions reveals a significant difference in the analysis of the negative mean (Table **3**). In the ARP group, there was a statistically significant smaller reduction of the observed soft tissue contour. However, the smaller distribution width of the values is unusual in the ARP group, except for the mean negative deviations.

## 4 DISCUSSION

This trial was performed with the specification that post-extraction changes of the soft-tissue contour surrounding the maxillary alveolus should be compared with a healing process without external influences as a baseline. The outcome in this study revealed a statistically significant difference in the soft-tissue contour at 6 (+/- 1) weeks post surgery in comparison to an untreated alveolus or post insertion of a collagen material into the alveolus in the sub-group of premolars. In these cases, the mean loss of soft tissue volume after use of the collagen material was significantly lower, but only of minor clinical relevance. There was also a trend revealed of a lower distribution width of the soft tissue changes in the ARP group. No explanation can be derived from the available data for the different outcomes in correlation with the tooth location. The hypothesis that the application of the collagen material leads to a stabilization of the soft tissue post tooth extraction could, therefore, be confirmed with reservations. Many studies investigated different materials and techniques for ARP. The studies vary widely, particularly in their methodology. There are few standards for the measurement of the soft tissue in particular. In this respect, the group of Vanhoutte V. *et al*. [25] developed a measurement method that is comparable with this study. They also investigated three-dimensional computer models from the digitalized models. However, they worked with a connective tissue transplant material that was used to close the alveolus. A relatively small sample size of 14 patients was studied over a period of three months. This measured positive effects, because there was no reduction of the soft tissue at the time of the final measurement. In the authors' opinion, the methods with digitalization and software analysis offer significant benefits in terms of reproducibility, and they facilitate high comparability compared to the measurement with probes by the individual administering the treatment and achieve a lower error rate and deviations due to measurement errors.

The time of the measurement has an effect on the assessment of the outcomes. It was expected that after 6 (+/-) weeks the bone healing was not yet complete and thus the focus was on the soft tissue remodeling processes [9].

Apart from a lack of standards for analysis and measurement, there is also often a lack of standards for the defect situation, which significantly impedes comparability. A useful classification, in this case, would be a classification by certain defect categories post tooth extraction. For example, this would be the number and state of the alveolus walls or the defect size itself. The thickness of the alveolar walls or, in the case of a molar extraction, for example, the interradicular bone septum, could also have a major effect on healing and regeneration and/or the ARP procedures [6]. However, under clinical conditions, the ability to determine the extent of the bone defect before surgery is limited. In the same way, it is almost impossible to reproduce the assessment of the alveolus in the context of a clinical trial. A CBCT image taken before tooth extraction could supply this information, but such imaging is generally ruled out for ethical reasons and to minimize radiation exposure. In this case, it would be indicated to use recommendations to improve the study quality for future research [26, 27]. A study by Karaca C. *et al.* [3] found a minor growth +0.06 and +0.25 mm on tissue three months post tooth extraction. In this case, a free connective tissue graft was used for coverage. However, the sample size in this study was only 10 patients. A further study confirmed this small reduction of the soft tissue contour post introduction of a free tissue graft [28]. A study with 15 patients using a connective tissue graft and DBBM-C (Demineralized, freeze-dried Bovine Bone Matrix with a 10% Collagen Proportion) also found only minor reductions up to 0.5 mm after a healing period of five months [29].

Despite the limited available data, there is at least a relatively large number of indications that ARP does not lead to a deterioration of the situation. Slight reductions of the resorption could also be achieved in some cases by using bioabsorbable membranes, calcium sulfate, xenogenous bone replacement materials or freeze-dried bones from human donors [12, 30].

This suggests that no one method can fully halt the resorption of the alveolar process, nor that any one method can be listed as particularly promising [31].

In general, the surgery without formation of a mucoperiosteal flap appears to be superior after an ARP with bovine replacement material and using a membrane, because in this case, more bone resorption was measured compared to the procedure with flap formation and exposed membrane [32].

A study in dogs and 3D measurement with a collagen material and DBBM (Demineralized, freeze-dried Bovine Bone Matrix) demonstrated that a minor shrinkage of the soft tissue contour could be achieved [33]. A further study with 40 subjects was able to demonstrate a significant difference in the reduction of resorption when using DBBM compared to beta-tricalcium phosphate (ß-TCP) and spontaneous healing [34]. At the moment, it must be assumed that ARP measures cannot entirely prevent a loss of the available soft tissue. However, it appears that appropriate measures can reduce the loss of soft tissue [35]. This requires further research and there is a need for procedures that are matched to the patient's individual situation and materials that are appropriate to the indication. One of the main indications for ARP would be the potential prevention of augmentations and thus additional interventions. Apart from the risks and side effects of additional surgery, this might also lead to improvements in the cost/benefit ratio and patient comfort.

The proposed hypothesis, which assumed that there is a difference in the preserved soft tissue between alveoli with and without application of a combination material consisting of a collagen cone and a collagen membrane can be accepted, with reservations, according to the outcomes of this study. The expectation of the hypothesis was an improved volume preservation of the soft tissue by implantation of the collagen cone. A statistically significant lower volume reduction could be found with ARP for premolars. However, this small dimensional change between the two treatment protocols is of little clinical relevance. It could also be demonstrated descriptively that the distribution width of the dimensional changes was lower with ARP. Thus, the use of ARP can facilitate the surgical procedure in individual cases and makes it easier to predict the outcome post extraction. Therefore, a recommendation in favor of using the material can be derived from the available data. Further research in this area remains useful and desirable because the reduction of the soft tissue loss post tooth loss constitutes an important contribution for rehabilitation with a prosthetic implant.

## ETHICS APPROVAL AND CONSENT TO PARTICIPATE

The study was approved by the committee (Ethics Committee of Ulm University, application no. 337/12, approved on 02/13/2013). The study was registered in the German Clinical Trial Register and the International Clinical Trials Registry Platform of the WHO as DRKS 00004769, date of registration: Feb. 28, 2013; and DRKS00005978, date of registration: Nov. 09, 2015.

## HUMAN AND ANIMAL RIGHTS

No animals were used in this research. All research procedures followed were in accordance with the ethical standards of the committee responsible for human experimentation (institutional and national), and with the Helsinki Declaration of 1975, as revised in 2008.

## CONSENT FOR PUBLICATION

Written and informed consent was obtained from the patients.

## Figures and Tables

**Table 1:** Distribution of the teeth by their region.

Region	Alveolar Ridge Preservation Group	Control Group	Total
Anterior teeth	14	15	29
Premolars	13	12	25
Molars	4	2	6

**Table 2:** Dimensional changes of the soft tissue of the total cohort in the Alveolar Ridge Preservation group (ARP) and the control group with information about the negative - maxima and minima of loss of dimension as well as the medians [unit: mm].

**Parameter**	**Measure**	**Valid Datasets**	**Maximum**	**Median**	**Minimum**	**Hypothesistest**
**Max negative**	ARP	31	-3.99	-2.52	-1.30	0.23
	Control	29	-5.00	-2.14	-1.02	
**Mean** **negative**	ARP	31	-1.56	-0.76	1.72	0.34
	Control	29	-1.59	-0.83	-0.30	
**Standard deviation**	ARP	31	0.29	0.54	1.24	0.52
	Control	29	0.25	0.57	1.59	

**Table 3:** Analysis of dimensional changes of the soft tissue in the subgroups “anterior teeth” and “premolars” with information about the negative maxima and miinimal as well as the negative median, standard deviations and statistic relevance [unit: mm]ARP: Alveolar Ridge Preservation.

**Parameter**	**Measure**	**Valid datasets**	**Maximum**	**Median**	**Minimum**	**Hypothesis Test**
**Anterior teeth**			-	-	-	-
**Max negative**	ARP	14	-3.99	-2.42	-1.36	0.17
	Control	15	-3.38	-2.10	-1.26	
**Mean** **negative**	ARP	14	-1.23	-0.73	-0,44	0.85
	Control	15	-1.29	-0.79	-0.54	
**Standard deviation**	ARP	14	0.29	0.50	0.89	0.59
	Control	15	0.26	0.54	1.07	-
**Premolars**			-	-	-	-
**Max** **negative**	ARP	13	-3.98	-2.69	-1.30	0.85
	Control	12	-5.00	-2.99	-1.02	
**Mean negative**	ARP	13	-1.20	-0.76	-0.32	**0.05**
	Control	12	-1.59	-0.96	-0.30	
**Standard ** **deviation**	ARP	13	0.32	0.59	1.24	0.65
	Control	12	0.25	0.66	1.59	
